# Psoas muscle area and paraspinal muscle fat in children and young adults with or without obesity and fatty liver

**DOI:** 10.1371/journal.pone.0259948

**Published:** 2021-11-17

**Authors:** Salman S. Albakheet, Mi-Jung Lee, Haesung Yoon, Hyun Joo Shin, Hong Koh

**Affiliations:** 1 Department of Radiology and Research Institute of Radiological Science, Severance Children’s Hospital, Yonsei University College of Medicine, Seoul, Korea; 2 Department of Radiology, King Faisal General Hospital, Al-Ahsa, Kingdom of Saudi Arabia; 3 Division of Gastroenterology, Hepatology and Nutrition, Department of Pediatrics, Severance Children’s Hospital, Yonsei University College of Medicine, Seoul, Korea; University of Houston, UNITED STATES

## Abstract

**Background:**

Little is known about the muscle condition in children with obesity.

**Objectives:**

To investigate the effect of obesity and fatty liver on muscle area and muscle fat in children and young adults.

**Materials and methods:**

We evaluated consecutive liver fat quantification MRIs in children and young adults between June 2015 and April 2019. We obtained hepatic fat and paraspinal muscle fat at mid L2 from the fat map, psoas muscle area (PMA) at mid L3, and z-score of PMA. The patient’s age, height and weight at the time of the MRI were recorded. Body mass index (BMI) z-score was also calculated. Spearman correlation and partial correlation analyses were performed. Univariate and multivariate regression analyses were also performed using significant variables.

**Results:**

A total of 132 patients (97 male) were included with a median age of 13.0 years (interquartile range 11–16 years). The median BMI was 23.7 kg/m^2^ (interquartile range 21.2–27.7 kg/m^2^). The weight, BMI, liver fat, and z-score of PMA were all higher in male patients than they were in female patients. The amount of liver fat had no correlation with muscle fat or PMA z-score after adjusting BMI. However, the BMI z-score was positively correlated with the PMA z-score (ρ = 0.432, p<0.001) even after adjusting for liver fat. On regression analyses, the BMI z-score had linear positive relationship with PMA z-score (β = 0.289, p<0.001) and muscle fat (β = 0.218, p = 0.016).

**Conclusions:**

Male children and young adults have greater PMA than do female children and young adults. Obesity is associated with higher PMA and paraspinal muscle fat. However, liver fat is not related with the muscle condition in children and young adults.

## Introduction

Over the past three decades, the prevalence of obesity in all age groups has been increased dramatically. Obesity has become a worldwide growing public health issue [1]. Since 1975, the worldwide obesity rate has approximately tripled according to the World Health Organization. More than 340 million children and adolescents aged 5 to 19 were overweight or obese in 2016. Accompanied by a worldwide increase in obesity, children and adolescent obesity has also increased [2]. Unfortunately, childhood obesity often continues into adulthood [3, 4]. Moreover, obesity in children and adolescents can cause serious obesity related comorbidities such as non-alcoholic fatty liver disease (NAFLD), obstructive sleep apnea, cardiovascular and metabolic disease including hypertension, dyslipidemia, low bone mineral density, and type 2 diabetes mellitus [5–8].

There are increasing concerns about the combined morbidity and mortality associated with obesity in adults, including sarcopenia [9, 10]. Sarcopenia is the progressive and generalized loss of skeletal muscle mass and a decrease in muscle strength or physical performance [11]. In adults, sarcopenia has been associated with the aging process, cardiometabolic disorders, and recently with sarcopenic obesity [12, 13]. Sarcopenia can also contribute to an increased prevalence of morbidity and mortality in pediatric patients [14–16]. In children and adolescents, sarcopenia has been linked with malnutrition and subsequent chronic liver diseases such as NAFLD, end-stage liver disease, and inflammatory bowel disease [16–19]. However, few studies have evaluated the effect of growth and obesity on muscle condition in children and young adults with or without NAFLD.

Therefore, the purpose of this study was to investigate the effect of obesity and fatty liver on muscle area and fat in children and young adults, including NAFLD patients.

## Materials and methods

### Study population

This retrospective cross sectional study with fully anonymized data was approved by the institutional review board of Severance hospital (IRB number, 4-2019-0394) and the need for written informed consent was waived. This study comprised consecutive pediatric patients and young adults who underwent fat quantification liver MRI examinations at our academic children’s hospital between June 2015 and April 2019. We reviewed the indications of the examinations. Patients were excluded if they had a known hepatic mass, liver disease (other than NAFLD), or a chronic inflammatory disease. At the time of the MRI examinations, the patients’ age, sex, weight, and height were recorded. Body mass index (BMI) was calculated using the following formula: weight (kg) / height^2^ (m^2^). BMI percentile and z-score was also calculated using the Age-based Pediatric Growth Reference Charts (http://www.bcm.edu/bodycomplab/BMIapp/BMI-calculator-kids.html). Overweight patients were defined by a BMI greater than the 85th percentile for age. Obesity was defined as a BMI at or above the 95th percentile for children of the same age and sex.

### MRI examination

During MRI examination, all patients were positioned supine with a 32-channel body coil over their abdomen. Patients were scanned in a 3T MRI system (Discovery 750w, GE Healthcare, Waukesha, WI, USA). MRI acquisition included single shot fast spin echo T2-weighted axial and coronal images. There was iterative decomposition of water and fat using echo asymmetry and least-squares estimation quantification (IDEAL-IQ) axial images of the liver. The anatomic location identifications of the liver, paraspinal muscles and psoas muscles were performed using single shot fast spin echo images. Through acquisition of a single breath hold, the proton density fat fraction maps of the abdomen (including the whole liver) were obtained using the IDEAL-IQ sequence with the following parameters: repetition time, 5.8 msec; field of view, 35–42 cm; image matrix sizes, 128 × 128; bandwidth, 125 kHz; flip angle, 3°; section thickness, 8 mm; and a single three-dimensional image with 25 to 30 sections.

### Image analysis

By using a commercially available picture archiving and communications system workstation (Centricity® Radiology RA1000, GE Healthcare, Barrington, IL, USA), all of the images were digitally reviewed and analyzed retrospectively by a radiologist with 6 years of experience. The radiologist was blinded to the medical history, clinical findings and the patients’ final diagnosis. In three contiguous images, oval or circular shaped maximum area of regions of interest (ROIs) were drawn in the right hepatic lobe. These ROIs offered a wide margin to the liver boundary, fissures, gall bladder fossa, artifacts and large blood vessels to assess the hepatic fat fractions. The ROIs with the maximal area were drawn in the paraspinal muscles bilaterally to assess the muscle fat fraction at the mid-level of L2. The mid-level of L2 was defined as the lowest level of the routine liver fat map in most patients. The average value of the fat fraction measurements of the liver and paraspinal muscles in each patient was calculated and used as the representative value of the patient. The normal limit of hepatic fat fraction was 6% [20].

**Fig 1:**
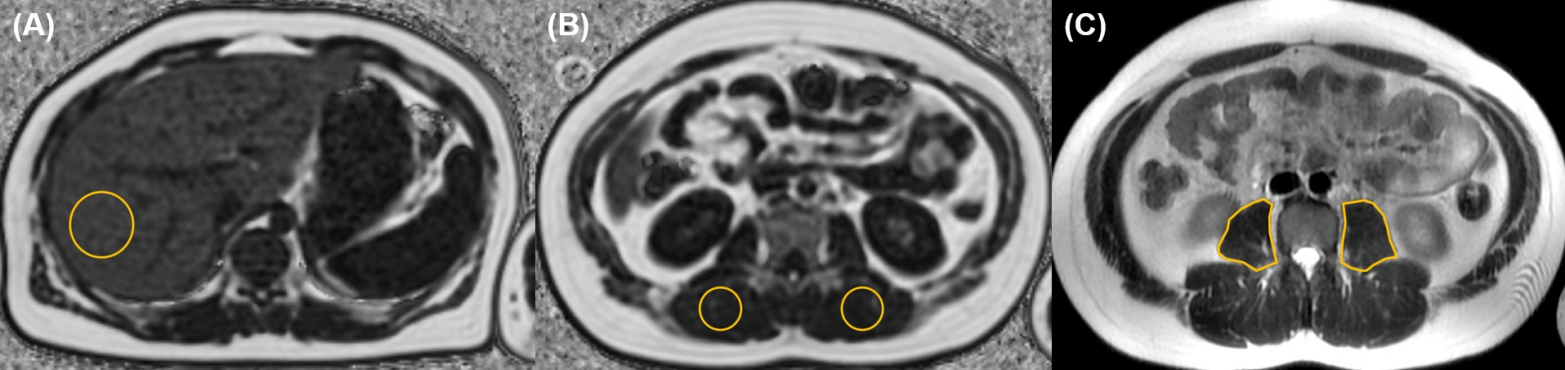
A 14-year-old boy with obesity (body mass index, 28.9 kg/m^2^) and fatty liver. From the liver MRI, (A) fat fraction of the liver (18%) was obtained from oval or circular region-of-interest (ROI, yellow circle) in the right lobe on the fat map. (B) The ROIs (yellow circles) for paraspinal muscle fat fraction were drawn bilaterally at the level of mid L2 and the mean value was 3.2% in this case. (C) In order to measure the psoas muscle area (PMA), free hand ROIs (yellow lines) were drawn at the level of mid L3 on T2-weighted axial images. The PMA was 1197.7 mm^2^ and the z-score of PMA was -2.56 in this patient.

**Fig 2:**
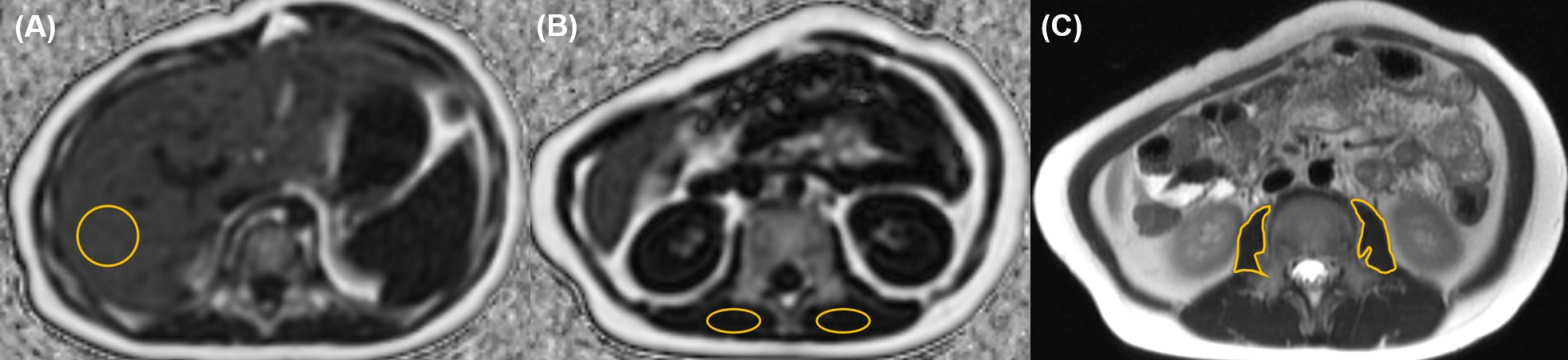
A 7-year-old boy with normal body mass index (18.3 kg/m^2^) and fatty liver. From the liver MRI, (A) fat fraction of the liver was 18.5%. (B) The mean paraspinal muscle fat fraction of the two circles was 2.1%. (C) The PMA was 265.1 mm^2^ and the z-score of PMA was -4.3 in this patient.

The psoas muscle cross-sectional area (PMA) was evaluated on a single slice T2 axial image at the mid-level of L3 (Figs 1 and 2) and the z-score of PMA was calculated [21]. Because the analytical age of the reference program is up to 16, the calculation in patients older than 17 years was based on the age of 16.

### Statistical analysis

All statistical analyses were performed using the SPSS software package (IBM SPSS Statistics version 21; IBM Corp., Armonk, NY, USA) and MedCalc Statistical Software (version 19.3.1; MedCalc Software Ltd, Ostend, Belgium). After normality test of Shapiro-Wilk, the Mann-Whitney U test was used for group comparison with continuous variables. The Spearman’s correlation coefficient (ρ) was calculated to evaluate the correlations between variables. In addition, partial correlation analyses were performed by adjusting BMI or liver fat. Univariate and multivariate analyses associated with PMA were also performed using significant variables. P-values < 0.05 were considered statistically significant.

## Results

### Patient characteristics and imaging measurements

Among the total 235 patients who underwent liver fat quantification MRI during the study period, 103 patients were excluded for underlying liver disease (other than NAFLD) or a limited field of view on liver MRI (that did not include the L3 level to measure PMA). Ultimately, 132 patients (97 male) were included in this study. The median age was 13.0 years with an interquartile range (IQR) of 11–16 years. The IQR of patients’ height was 147–164 cm, and that of weight was 47–70 kg. The IQR of BMIs was 21.2–27.7 kg/m^2^ with a median BMI of 23.7 kg/m^2^. Based on the calculated BMI percentile, 29 patients (22%) were overweight and 56 patients (42%) were obese.

The median hepatic fat fraction was 18.0% with the IQR of 5.9–32.9%. The IQR of paraspinal muscle fat fraction was 1.9–3.3% with a median of 2.5%. The IQR of PMA was 460.0–810.9 mm^2^ with a median of 601.8 mm^2^. The median z-score of PMA was -3.60 (Table 1).

**Table 1:** Demographics and group comparisons.

All patients							
	**All (n = 132)**		**Male (n = 97)**		**Female (n = 35)**		P-value*
	Median	Interquartile range	Median	Interquartile range	Median	Interquartile range	
**Age (years)**	13.0	11.0–16.0	13.0	11.0–16.0	16.0	11.0–17.0	0.075
**Height (m)**	1.57	1.47–1.64	1.57	1.45–1.68	1.57	1.49–1.60	0.473
**Weight (kg)**	58.0	47.3–70.0	60.0	49–73	52.0	42.0–64.0	**0.020**
**BMI (kg/m** ^**2**^ **)**	23.7	21.2–27.7	24.3	21.7–27.8	22.1	18.0–26.0	**0.006**
**Liver fat (%)**	18.0	5.9–32.9	20.1	10.9–34.0	10.9	3.1–20.0	**0.005**
**Paraspinal muscle fat (%)**	2.5	1.9–3.3	2.3	1.8–3.3	2.5	2.2–3.4	0.247
**Psoas muscle area (mm** ^**2**^ **)**	601.8	460.0–810.9	663.9	514.1–869.5	467.0	353.5–588.8	**<0.001**
**Psoas muscle area z-score**	-3.60	-4.03 –-3.14	-3.57	-3.91 –-3.06	-3.93	-4.48 –-3.44	**0.002**
**Normal group**							
	**All (n = 29)**		**Male (n = 17)**		**Female (n = 12)**		P-value*
	Median	Interquartile range	Median	Interquartile range	Median	Interquartile range	
**Age (years)**	16.0	13.5–19.0	16.0	14.0–19.5	16.0	12.3–17.0	0.263
**Paraspinal muscle fat (%)**	2.2	1.5–2.6	1.9	1.2–2.4	2.5	2.2–2.8	**0.014**
**Psoas muscle area (mm** ^**2**^ **)**	514.6	374.5–686.1	585.8	437.4–900.0	409.3	354.1–534.9	**0.027**
**Psoas muscle area z-score**	-4.04	-4.43 –-3.66	-3.89	-4.19 –-3.52	-4.28	-4.57 –-3.75	**0.043**
**Fatty liver group**							
	**All (n = 99)**		**Male (n = 77)**		**Female (n = 22)**		P-value*
	Median	Interquartile range	Median	Interquartile range	Median	Interquartile range	
**Age (years)**	13.0	11.0–16.0	12.0	11.0–15.0	15.5	10.0–17.0	0.116
**BMI z-score**	1.70	1.20–2.10	1.80	1.40–2.20	1.25	0.40–1.83	**0.002**
**Liver fat (%)**	25.0	16.0–35.0	28.0	17.4–35.6	18.0	12.6–29.3	**0.025**
**Paraspinal muscle fat (%)**	2.6	2.0–3.4	2.7	2.0–3.4	2.5	2.1–3.6	0.705
**Psoas muscle area (mm** ^**2**^ **)**	632.1	478.0–836.0	663.9	519.2–885.4	478.8	345.8–719.1	**0.003**
**Psoas muscle area z-score**	-3.56	-3.90 –-3.06	-3.55	-3.84 –-3.01	-3.57	-4.42 –-3.28	0.143

BMI body mass index.

*Comparisons between male and female groups using Mann-Whitney U test.

### Sex comparison and group population

When comparing between male and female patients, there were no differences in age, height or paraspinal muscle fat. However, the weight (p = 0.020), BMI (p = 0.006), liver fat (p = 0.005), and z-score of PMA (p = 0.002) were higher in male patients than they were in female patients (Table 1).

Twenty-nine patients had both normal BMIs and liver fat fraction, including 17 male patients and 12 female patients at an age range of 7–24 years. The range of paraspinal muscle fat was 1.0–7.7% with the median of 2.2%, while that of PMA was 210.3–1227.3 mm^2^ with the median of 514.6 mm^2^. The range of z-score of PMA was -4.65 to -2.98 with the median of -4.04 in this normal group. The paraspinal muscle fat (p = 0.014) was lower and PMA (p = 0.027) or z-score of PMA (p = 0.043) was higher in male than in female in the normal group (Table 1).

In the fatty liver group (n = 99), the male patients (n = 77) had higher BMI z-score (p = 0.002), liver fat (p = 0.025), and PMA (p = 0.003) values than did the female patients. Age and paraspinal muscle fat were not different between male and female patients in the fatty liver group (p > 0.05, Table 1).

### Correlation results between muscle condition, obesity and fatty liver

Table 2 summarizes the correlation results between the muscle condition and obesity or fatty liver. The BMI z-score showed consistent positive correlations with the z-score of PMA even after adjusting for liver fat (ρ = 0.432, p < 0.001). The liver fat fraction also was positively correlated with the z-score of PMA and paraspinal muscle fat fraction in all patients. However, after adjusting for BMI, the liver fat was not correlated with the PMA or paraspinal muscle fat.

**Table 2:** Correlation between muscle condition and liver fat or body mass index.

		Psoas muscle area z-score		Paraspinal muscle fat (%)	
		ρ	p-value	ρ	p-value
**BMI z-score**	**All patients**	**0.511**	**<0.001**	**0.236**	**0.006**
	**Adjusting liver fat**	**0.432**	**<0.001**	0.138	0.115
**Liver fat (%)**	**All patients**	**0.330**	**<0.001**	**0.244**	**0.005**
	**Adjusting BMI**	-0.001	0.992	0.059	0.507

BMI body mass index.

### Regression results

**Fig 3:**
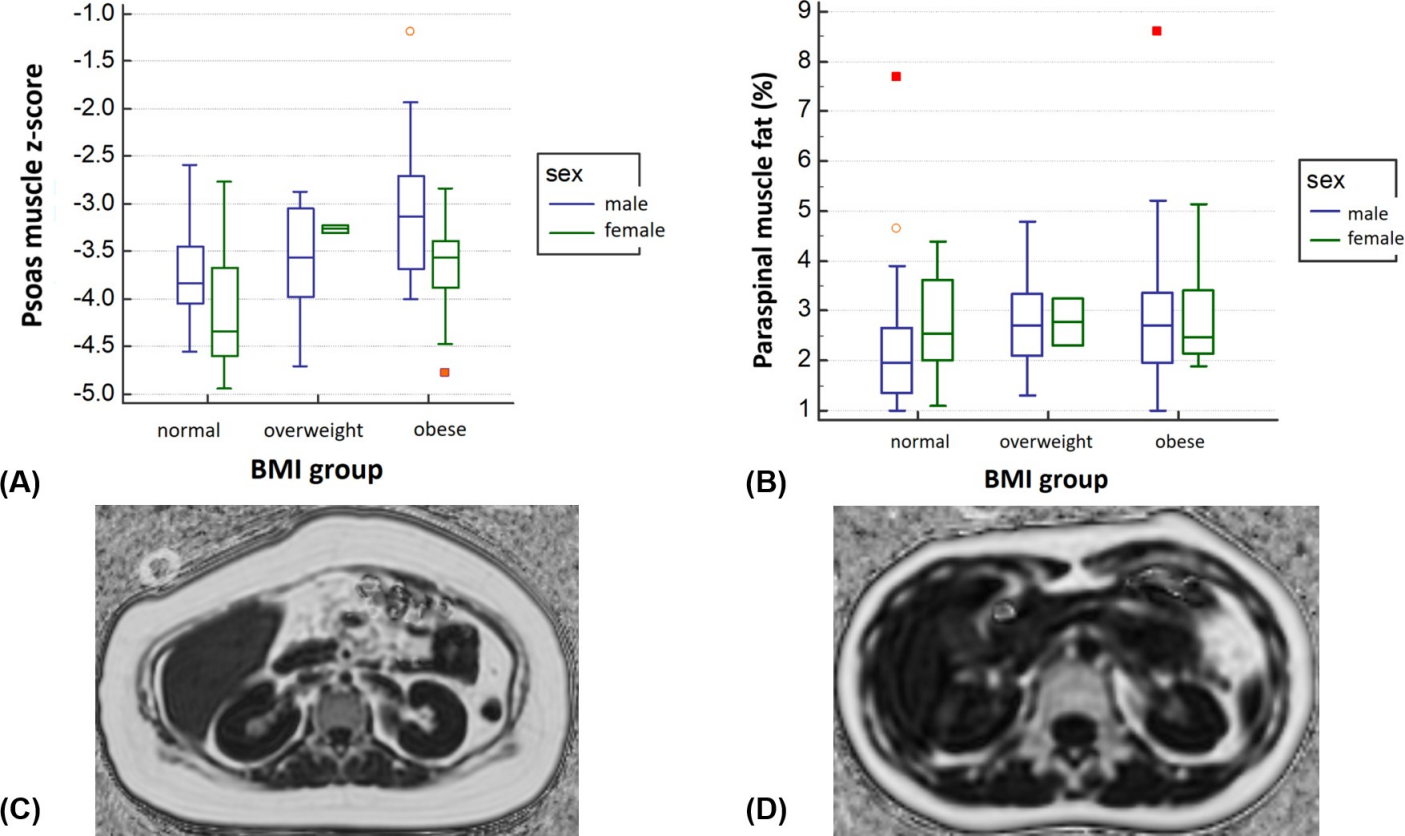
Box plots and representative images for the group comparison of sex and body mass index (BMI) groups. Based on BMI, 47 patients were normal, 29 were overweight, and 56 were obese. (A) Z-score of PMA was increased according to the increased BMI groups. Male patients had higher z-score of PMA than did female patients in all BMI groups. (B) The paraspinal muscle fat fraction also showed increasing tendency according to the increased BMI groups; however, the sex difference was not definite. (C) A 13-year-old female with obesity (BMI, 32.4 kg/m^2^) and fatty liver (10.9%) had 5.2% paraspinal muscle fat and 595.2 mm^2^ PMA (z-score, -3.33). (D) A 15-year-old female without obesity (BMI, 15.8 kg/m^2^) nor fatty liver (3%) had 1.9% paraspinal muscle fat and 355.9 mm^2^ PMA (z-score, -4.48).

**Fig 4:**
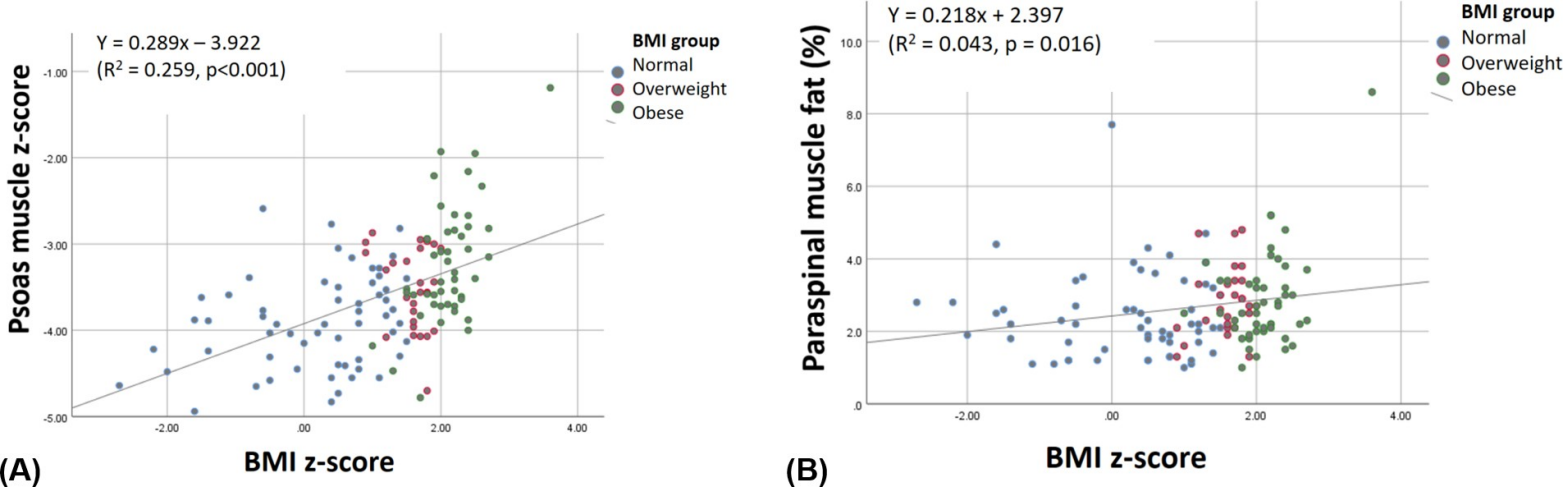
Scatter plots for the correlations between body mass index (BMI) z-score and psoas muscle area z-score or paraspinal muscle fat. BMI z-score has positive linear correlations with (A) z-score of PMA (β = 0.289, p < 0.001) and (B) paraspinal muscle fat (β = 0.218, p = 0.016). The dot color means the BMI group as normal with blue, overweight with red, and obese with green.

Regression analyses for PMA were performed only for z-score of PMA, because PMA is associated with body size and growth in pediatric patients. Univariate analysis showed that age had negative association with z-score of PMA (β = -0.056, p < 0.001). Sex (male, β = 0.468, p < 0.001), BMI z-score (β = 0.289, p < 0.001), and liver fat (β = 0.014, p = 0.001) were also associated with z-score of PMA (Table 3, Figs 3 and 4). Multivariate regression analysis also demonstrated that sex (male, β = 0.239, p = 0.044) and BMI z-score (β = 0.223, p < 0.001) had positive linear relationships with the z-score of PMA (R^2^ = 0.297), even though age and liver fat did not.

**Table 3:** Regression analyses for psoas muscle z-score in consideration with other variables.

		Univariate regression analysis			Multivariate regression analysis		
		Estimate (β)	Standard error	p-value	Estimate (β)	Standard error	p-value
**Psoas muscle area z-score**							
	**Age (years)**	-0.056	0.014	**<0.001**	-0.024	0.014	0.078
	**Sex**						
	**Female**	Reference			Reference		
	**Male**	0.468	0.123	**<0.001**	0.239	0.118	**0.044**
	**BMI z-score**	0.289	0.043	**<0.001**	0.223	0.058	**<0.001**
	**Liver fat (%)**	0.014	0.004	**0.001**	-0.001	0.004	0.889
**Paraspinal muscle fat**							
	**Age (years)**	-0.016	0.027	0.540	0.017	0.029	0.580
	**Sex**						
	**Female**	Reference			Reference		
	**Male**	-0.106	0.238	0.655	-0.368	0.250	0.143
	**BMI z-score**	0.218	0.090	**0.016**	0.247	0.124	**0.048**
	**Liver fat (%)**	0.014	0.007	0.052	0.007	0.009	0.451

BMI body mass index.

When considering the paraspinal muscle fat as a parameter of the muscle condition, both univariate and multivariate regression analyses found that only the BMI z-score was positively related to the paraspinal muscle fat (β = 0.218, p = 0.016 on univariate and β = 0.247, p = 0.048 on multivariate, Table 3 and Fig 4).

## Discussion

Considering the increasing incidence of childhood obesity, obesity-related diseases have become major concerns in developed countries. In our study, obesity in children and young adults was not associated with decreased PMA. On the contrary, obesity was associated with increased PMA. There was also increased paraspinal muscle fat in obese patients than there was among non-obese patients. However, fatty liver was not associated with the muscle condition in our study group. Both muscle volume and muscle quality (including strength) are important for the diagnosis of sarcopenia. Further long-term research with this topic is needed in the terms of childhood obesity.

The skeletal muscle volume increases in childhood at least until spinal maturity [22]. However, there is limited known about the normal range of skeletal muscle volume in children and adolescents. A recent study demonstrated that muscle mass has an increasing trend with age for both boys and girls aged 3–17 years [23]. This group also demonstrated that muscle mass increased slowly until 8–9 years, and then rapidly until age 14–15 years. Furthermore, the mean muscle mass was greater in boys than it was in girls at each age. Our study also demonstrated that male patients had higher PMA and z-score in the normal group than did female patients. We also evaluated z-score of PMA which considering not only age but also sex, and found that z-score of PMA was also higher in the male than in the female of normal group. Therefore, when analyzing sarcopenia, sex must be taken into account.

Sarcopenic obesity refers to the presence of both sarcopenia and obesity. This condition is mainly studied in middle-aged women and the elderly [24–26]. Prior work has focused on the pathogenic interaction between adipose tissue and muscle in sarcopenia [27]. However, there are debates regarding the relationship between sarcopenia and obesity [28]. Anorexia and underweight are recognized causes of sarcopenia [11]. On the one hand, however, sarcopenia increases the risk of obesity [29]. Therefore, obesity can exacerbate sarcopenia and vice versa (sarcopenic obesity). In addition, there are limited studies regarding sarcopenia in children [17]. Our study demonstrated that obesity has a positive relationship with PMA in children and young adults. These findings may be associated with the special situation of growth in childhood. However, the correlation between obesity and PMA was also maintained when the age and sex was corrected using z-score. Interestingly, as obesity increases, not only PMA, but also the amount of paraspinal muscle fat increases. Considering the patient’s health, the volume of muscles is important, but the quality of muscles is also important. Measurement of PMA from a single slice cross sectional image represents an easily accessible and quick method to assess the presence of sarcopenia. However, controversies exist on whether a single psoas muscle is sufficient to reflect other skeletal muscles. Moreover, muscle volume or fat change during growth cannot be determined in this cross sectional study. Therefore, further validation about our study is needed.

Both muscle mass and muscle fat were associated with patients’ prognosis in a study about liver transplantation [30]. The skeletal muscle fat accumulation is also associated with insulin resistance and type 2 diabetes in children [31]. However, muscle fat accumulation can be reversible. During a 1-year multidisciplinary intervention for childhood obesity, there was reduced fat content in the liver and skeletal muscle [32]. Therefore, monitoring muscle fat in children with obesity may be of benefit as a quantitative parameter.

There is an increasing number of fatty liver diseases in both adults [33, 34] and children [35]. In adults, NAFLD was associated with low muscle mass and low muscle strength [36, 37]. However, there is only one study that addresses the relationship between NAFLD and muscle mass in children with obesity [38]. This group performed dual-energy X-ray absorptiometry in the evaluation of relative muscle mass and demonstrated an independent association between low muscle mass and NAFLD in overweight/obese children. However, the diagnosis of fatty liver was only based on liver echogenicity, which is neither specific nor quantitative. Our study used proton density fat fraction, which is a widely used method, to quantify the amount of fat in the liver [39]. Based on this quantitative method, we found no relationship between fatty liver and PMA in our study group. Also, after adjusting BMI, liver fat was not associated with paraspinal muscle fat. Further studies are needed to evaluate the relationship between BMI and paraspinal muscle fat in fatty liver patients and determine when muscle mass reduction occurs in pediatric fatty liver patients in the long term.

There are several limitations in this study. First, it was retrospective in design. We used liver MRI for the evaluation of psoas muscle at the level of L3. Many patients were excluded because the L3 level was not included. Therefore, there may have been a selection bias towards patients with hepatomegaly. In addition, we could not evaluate the muscle fat in the psoas muscle from the limited field of view of liver fat map. There is no report about fat infiltration preference or pattern in diffuse metabolic disease condition. So we selected the psoas muscle which was included in the field of view of our liver MRI and has the z-score values for children and adolescents. And there was no grossly different fat accumulation in psoas muscle and paraspinal muscle in our patients. However, additional studies with more muscle volume, including paraspinal muscles, are needed to validate our results. The second limitation is that it is not a long-term study, but a cross sectional study. Therefore, it is not possible to evaluate how these results will affect patients’ outcome. Long-term cohort research is needed. There was a lack of analysis of the other obesity-related complications or diseases. While it was not the focus of this study, it is necessary to evaluate whether muscle conditions are related to other cardiometabolic complications. Lastly, the z-score of PMA of our study subjects was generally low. It may be due to a race difference, but further research is needed.

In conclusion, male children and young adults have greater PMA than do female children and young adults regardless of obesity. Obesity is not only associated with greater PMA but also with higher paraspinal muscle fat. However, fatty liver was not associated with psoas muscle size or paraspinal muscle fat fraction in children or adolescents. Additional long term studies to determine the pathophysiological mechanisms that result in increased muscle fat in this age group are needed.

## Supporting information

S1 Data (XLSX)
